# *Lactococcus lactis* Delivery of Surface Layer Protein A Protects Mice from Colitis by Re-Setting Host Immune Repertoire

**DOI:** 10.3390/biomedicines9091098

**Published:** 2021-08-29

**Authors:** Ananta Prasad Arukha, Christian Furlan Freguia, Meerambika Mishra, Jyoti K. Jha, Subhashinie Kariyawasam, Neil A. Fanger, Ellen M. Zimmermann, Gary R. Fanger, Bikash Sahay

**Affiliations:** 1 Department of Infectious Diseases and Immunology, University of Florida, Gainesville, FL 32608, USA; ananta.arukha@ufl.edu (A.P.A.); Meerambika.mishra@gmail.com (M.M.); 2 Comparative, Diagnostic and Population Medicine, University of Florida, Gainesville, FL 32608, USA; skariyawasam@ufl.edu; 3 Rise Therapeutics, Rockville, MD 20850, USA; cfreguia@risetherapeutics.com (C.F.F.); jjha@risetherapeutics.com (J.K.J.); gfanger@risetherapeutics.com (G.R.F.); 4 Virtici, Seattle, WA 98122, USA; nfanger@virtici.com; 5 Division of Gastroenterology, University of Florida College of Medicine, Gainesville, FL 32608, USA; ezimmermann@ufl.edu

**Keywords:** colitis, *Lactococcus*, microbiome

## Abstract

Inflammatory bowel disease (IBD) is characterized by gastrointestinal inflammation comprised of Crohn’s disease and ulcerative colitis. Centers for Disease Control and Prevention report that 1.3% of the population of the United States (approximately 3 million people) were affected by the disease in 2015, and the number keeps increasing over time. IBD has a multifactorial etiology, from genetic to environmental factors. Most of the IBD treatments revolve around disease management, by reducing the inflammatory signals. We previously identified the surface layer protein A (SlpA) of *Lactobacillus acidophilus* that possesses anti-inflammatory properties to mitigate murine colitis. Herein, we expressed SlpA in a clinically relevant, food-grade *Lactococcus lactis* to further investigate and characterize the protective mechanisms of the actions of SlpA. Oral administration of SlpA-expressing *L. lactis* (R110) mitigated the symptoms of murine colitis. Oral delivery of R110 resulted in a higher expression of IL-27 by myeloid cells, with a synchronous increase in IL-10 and cMAF in T cells. Consistent with murine studies, human dendritic cells exposed to R110 showed exquisite differential gene regulation, including IL-27 transcription, suggesting a shared mechanism between the two species, hence positioning R110 as potentially effective at treating colitis in humans.

## 1. Introduction

Inflammatory bowel disease (IBD) is a chronic intestinal ailment affecting 6.8 million individuals globally [1]. In the United States, 77 children and 478 adults per 100,000 are affected by the disease [2]. Typically, IBD is divided into two major groups: Crohn’s disease (CD) and ulcerative colitis (UC). In UC, the intestinal inflammation is typically superficial, involving only the mucosal layer, and confined to the colon. In CD, the inflammation is transmural, extending through the intestinal wall to the serosal layer, and can affect the entire gastrointestinal (GI) tract. The distal ileum and ascending colon are the most common sites of involvement. While the significant area of pathology (and the most responsible for the patients’ symptoms) is the GI tract, inflammatory mediators can also leak into the systemic circulation and affect other organs, such as the musculoskeletal system [3,4,5,6], and their disease progression is linked to the gut pathology. The systemic inflammation ameliorates with improvement of GI health [7,8]. Treatment of IBD includes untargeted therapies (e.g., amino-salicylates and glucocorticoids) and targeted biologics (e.g., anti-tumor necrosis factor antibodies), signaling inhibitors (e.g., Janus kinase inhibitors), or lymphocyte trafficking modulators (e.g., anti-α4β7 integrin antibodies). Unfortunately, none of these therapies are effective in all the patients, and biological therapies often fail over time, due to immunogenicity. These therapies can also result in severe side effects and toxicities, stressing the importance of identifying and developing new IBD therapies.

Gut microbiota plays a critical role in the homeostatic maintenance of health, and commensal bacteria function to maintain integrity of the intestinal epithelial barrier, as well as regulate innate and adaptive immune cell function [9]. Perturbation of the gut microbiome is associated with several diseases, including IBD. IBD patients’ gut microbiome is characterized by dysbiosis and leaky gut [10]; hence, there is an opportunity to reinstate the gut microbiota as a treatment for IBD. Several attempts have been made to overhaul the microbial components using fecal microbial transfer (FMT) treatment, which provided good protective responses in some cases; however, safety is an issue with FMT, as there is always a chance to introduce antibiotic-resistant bacteria and other unwanted microbes or pathogens in the recipients [11,12]. As an alternative to FMT, several groups have used isolated single bacterial species or a consortium of selected bacterial species to reduce GI inflammation and manage colitis. Overall, all these strategies have not yielded consistently positive results and based upon the limitations in our understanding of the mechanism by which these bacterial strains work in vivo, it has been difficult to clinically progress from the failures and successes of these studies [13]. Thus, a more selected targeting approach to modulate immune pathways associated with the microbiome with better understood and more fully defined mechanisms of action, could yield a promising new strategy to mitigate the devastating effects of gut inflammation. A promising and potentially safer approach to the treatment of IBD is to leverage the body’s own natural immune regulatory mechanisms that reside in the interactions between commensal microbial products with host immune cells lining the gut epithelial layer.

We and others have recently identified a handful of molecules that could be used as biotherapeutics for IBD and delivered them at the colonic mucosa [14,15]. Specifically, we discovered that the oral delivery of surface layer protein A (SlpA) exerts regulatory signals that result in the mitigation of colitis, maintenance of healthy gastrointestinal microbiota, and protection of the gut mucosal barrier function [15,16,17]. Delivery of purified SlpA bears significant hurdles, including poor protein solubility, manufacturing and formulation challenges, and the cost of orally delivering such a biologic in humans is economically prohibitive. A better strategy to deliver SlpA orally is via a safe, food-grade probiotic, such as *Lactococcus lactis,* engineered to stably express SlpA. To create this therapy, the *SlpA* gene was inserted into the genome of *L. lactis* by replacing the thymidylate synthase (*thyA*) gene, thus providing a containment strategy, such that the organism will not grow after being excreted from the body. Additionally, SlpA was engineered as a secretory protein to increase the pharmacodynamic engagement of intestinal-associated immune cells.

Here, we build upon our previous data to confirm that the oral administration of SlpA expressing *L. lactis* protects mice from T cell-induced colitis and advances our mechanistic understanding of how SlpA influences immunological changes to mitigate gut inflammation. Our results showed that SlpA-expressing bacteria lead to an enrichment of beneficial bacteria in the gut of mice and reduce inflammatory mediators, such as IL-17. Additionally, we report an increase in IL-10 expression and the transcription factor cMAF in colonic T cells after SlpA administration in murine IBD models. A common immunological regulation was observed using primary human dendritic cells, stressing the translational potential of this novel IBD treatment strategy.

## 2. Materials and Methods

### 2.1. Generation of R110

The SlpA expression cassette was integrated in the *L. lactis* genome using standard double homologous recombination methods [18,19]. To direct chromosomal integration, we synthesized DNA encoding the *slpA* sequence, flanked 5’ and 3’ by 500 bp overhangs, homologous to the upstream and downstream *thyA* target gene sequence region (Blue Heron Biotech, Bothell, WA, USA). SlpA-thyA was inserted into the multiple cloning site (MCS) of the pRISE1.2 plasmid (Rise Therapeutics, Rockville, MD, USA), which contains a temperature-sensitive origin of replication. Transformation of the final plasmid was performed by electroporation at 2000V (Eporator, Eppendorf, Enfield, CT, USA). Cell were initially grown at 30 °C, and then the temperature was shifted to 37 °C to select for integrant cells. The *thyA* homologous sequences allowed for stable *slpA* insertion at the *thyA* locus via a double crossover event, replacing *thyA* from the *L. lactis* chromosome. The purpose of targeting and deleting the *thyA* gene is to allow for containment; that is, the SlpA expressing *L. lactis* will not propagate without thymidine supplementation [20]. A positive selection system was utilized to identify clones containing the integrated *slpA* expression cassette, as previously described [19,21]. For positive selection, M17 medium (Millipore Sigma, Burlington, MA, USA) was supplemented with 4µg/ml of the dihydrofolate reductase (DHFR) inhibitor trimethoprim and 20 µg/ml of thymidine (Millipore Sigma, Burlington, MA, USA). In the presence of trimethoprim, wild-type *L. lactis* containing a functional *thyA* led to tetrahydrofolate (THF) pool depletion and cell death through the inhibition of numerous THF-dependent reactions. However, inactive *thyA* (via *slpA* cassette integration) allowed THF levels to remain high, even in the presence of trimethoprim, thus, *L. lactis* containing a stably integrated SlpA expression cassette grew on minimal media supplemented with trimethoprim and thymidine. Stability of the integration event was confirmed by growing the selected clones under non-selective conditions in M17 media supplemented with 20 µg/ml thymidine for 100 generations. The selected clone was named R110.

### 2.2. Growth and Maintenance of R110

R110 was grown in M17 media supplemented with 20 µg/mL thymidine. The bacterial stocks were made with 15% glycerol, and the tubes were frozen in −80 °C. 

### 2.3. Mice

Eight-week-old Rag1^−/−^ (Jackson Laboratory; Stock # 002216; Bar Harbor, ME, USA) and C57BL/6 (Jackson Laboratory Stock # 00664; Bar Harbor, ME, USA) were housed in the Animal Care Facility at the University of Florida, Gainesville. Food and water were provided ad libitum.

### 2.4. Ethical Animal Care

Animal procedures were approved by the Institutional Animal Care and Use Committee of the University of Florida, IACUC protocol 201810263. All the experiments were carried out in AAALAC accredited Animal Care Facility at the University of Florida, under the supervision of veterinarians specialized in laboratory animal medicine. 

### 2.5. T Cell Colitis Induction

For the initiation of colitis via CD45RB^Hi^ CD4^+^ T cells transplantation into Rag1^−/−^ mice, spleen cell suspensions, obtained from healthy C57BL/6 mice, were pooled, and CD4^+^ T cells were isolated using EasySep™ Mouse CD4^+^ T Cell Isolation Kit (Stemcell Technology; Cat # 19852; Vancouver, BC, Canada). Isolated CD4^+^ T cells were stained with anti-CD45RB (Clone C363-16A), -CD3 (Clone 17A2), -CD4 (Clone GK1.5), and a Zombie Violet™ Fixable Viability Kit (Biolegend, San Diego, CA, USA) before sorting them on a SONY SH800 cell sorter. Viable CD45RB^HI^ cells were sorted and washed with PBS before injecting into the 8-week-old Rag1^−/−^ mice. All mice were injected with 5 × 10^5^ cells/mouse via the intraperitoneal route. 

### 2.6. Fecal Occult Blood Determination

Fecal occult blood was determined by using Hemoccult Sensa (Cat # 64151; Beckman Coulter, Inc. Brea CA, USA). Mouse feces were smeared over the paper box provided in the kit and left for three days at room temperature. A drop of the developer was added onto the smeared feces, and the color development was noted after 30-s. An arbitrary grade was decided, based on the range from no color (0) to dark blue (4), to determine the level of fecal occult blood, as performed previously [15]. 

### 2.7. FITC Dextran Assay

Passive transepithelial absorption of FITC-labeled dextran (Sigma-Aldrich, St. Louis, MO, USA) in vivo was used to determine intestinal barrier function, as previously described [15]. Mice were gavaged with FITC-dextran, MW 4000 (60 mg/100 g body weight). Blood was collected retro-orbitally after proper anesthetization. Fluorescence intensity in the serum was measured with a fluorimeter (BioTek Synergy HTX multi-mode microplate reader; Winooski, VT, USA). FITC-dextran concentrations in the mouse sera were determined from standard curves, generated by serial dilution of FITC-dextran, using blank subtraction in the test samples and sera from mice that were not gavaged with the permeability tracer.

### 2.8. Fecal Albumin Assay

Murine feces were dissolved in dilution buffer (50 mM Tris, 0.14 M NaCl, 0.05% Tween 20, pH 8.0) to a concentration of 100 mg/ml. Albumin contents were measured using the mouse albumin ELISA Kit (Cat # E99-134; Bethyl Laboratory, Montgomery, TX, USA), according to the manufacturer’s instructions. 

### 2.9. Isolation of Colonic Lamina Propria Cells

Freshly isolated colons were washed with PBS and then incubated with 15 mM EDTA solution in PBS for 30 mins on ice to remove epithelial cells. After a rinse with PBS, 0.5 cm long pieces of colon were digested with Collagenase VIII (250 μg/mL) and DNAseI (150 μg/mL) for 90 mins at 37 °C in 5% CO_2_. The digested tissues were washed with cold PBS and resuspended in 40% percoll and 80% percoll underlay before centrifugation at 1300× *g* for 20 mins at room temperature. After removing the top fat- and epithelial cell-rich sections, cells in the upper layer were collected, washed, and stained for flow cytometry.

### 2.10. Flow Cytometry Analysis of Isolated Cells

Colonic lamina propria cells were stained with a Zombie UV fixable stain kit (Biolegend, San Diego, CA, USA). Washed cells were incubated with mouse Fc blocking reagent (Miltenyi Biotec, Auburn, CA, USA), as per the manufacturer’s instructions, before staining with combinations of the following antibodies (or their corresponding isotype controls): CD45 (30-F11), CD3 (145-2C11), CD4 (RM4-5), IL-17A (TC11-18H10.1)/Rat IgG1, κ, IL-10 (JES5-16E3)/Rat IgG2b, κ, RORγt (AFKJS-9)/Rat IgG2a, κ, IL-27 (355025) /Rat IgG2a, and cMAF (sym0F1)/ Rat IgG2b κ. To detect intracellular cytokines, cells were fixed and permeabilized with BD Cytofix/Cytoperm (BD Biosciences, San Jose, CA, USA). Colonic T cells were stimulated with a cell activation cocktail containing Brefeldin A (Biolegend, San Diego, CA, USA) for 3 h. The Transcription Factor Fixation/Permeabilization Kit (Thermo Fisher Scientific, Grand Island, NY, USA) was used for RORγt and cMAF staining. After staining, a BD LSRFortessa (BD Biosciences, San Jose, CA, USA) cell analyzer was used to acquire fixed cells. Data were analyzed with FlowJo software (Tree Star, Ashland, OR, USA). Antibodies, and their corresponding isotype controls, were purchased from eBioscience (San Diego, CA, USA), Biolegend (San Diego, CA, USA), BD Pharmingen, or R&D Systems (Minneapolis, MN, USA).

### 2.11. Microbial Composition Analysis

Mice feces were sent to Novogene Corporation Inc. (Sacramento CA, USA) for microbiota analysis. The total fecal DNA were isolated and amplified using the primers specific for the V3 and V4 regions of the 16s rRNA gene. The amplicons were sequenced using an Illumina high-throughput sequencer with paired-end sequencing strategy. The sequences were saved in FASTQ format for further analysis. The obtained sequences were identified from phylum to species levels using the Silva database (version 2017.12).

### 2.12. Quantitative Real-Time PCR

Total RNA was isolated from the tissues or the cells using a combined protocol with Trizol and Aurum Total RNA Mini kit (Cat# 7326820, BioRad, Hercules, CA, USA). The concentration and purity of RNA samples were determined by measuring the absorbance (A260/A280) using a NanoDrop 2000 spectrophotometer (Thermo Fisher Scientific, Grand Island, NY, USA). A total of 0.5 μg of RNA was converted into cDNA using Superscript III (Invitrogen Corporation, Carlsbad, CA, USA). The cDNA was used as the template for qPCR analysis. For the final 10 μL reaction, 0.1 μL of cDNA was mixed with 5 μL of SYBR green and 10 pmol of both forward and reverse primers. The qPCR was performed on a MIC PCR platform, and differences in the transcripts were analyzed using the threshold cycle (2^−ΔΔCT^) method.

### 2.13. Murine Dendritic Cells Experiment

Bone marrow from mice were isolated by flushing the femur and tibia. The bone marrow cells were cultured with 20 ng/mL of murine GMCSF (Cat # 713704, Biolegend, San Diego, CA, USA) for 18 h. Later, the suspended cells were removed, and fresh media with GMCSF was added to the attached cells. Three days after initiation, fresh GMCSF was added to the culture. After a week, cells in suspension were collected, washed, and stimulated with purified SlpA.

### 2.14. Human Dendritic Cells Experiment

Human peripheral blood monocytes were isolated from two human donors using the lymphocyte separation medium (LSM, Corning Scientific, Tewksbury, MA, USA). Monocytes were isolated from PBMCs using CD14 MicroBeads (Miltenyi, Auburn, CA, USA). CD14^+^ monocytes were cultured in Mo-DC differentiation medium for seven days and in maturation medium for an additional three days. Human dendritic cells were co-incubated with the wild-type *L. lactis* or SlpA expressing *L. lactis* (R110) at a ratio of 1:1 overnight at 37°C. The supernatant was then used for analysis.

### 2.15. RNAseq Analysis

RNA-seq was performed using the paired-end sequencing, according to standard Illumina protocols by Novogen (Sacramento, CA, USA). The quality of RNA-Seq FASTQ data were checked using the FasQC program. Data passing the quality control were imported into the CLC Genomics Workbench (Version 20) for RNA-Seq analysis. The gene expression table was generated by using the *Homo sapiens* (hg38) genome as the reference sequence. Significant differential expression genes were determined with a selection threshold of *p*-value ≤ 0.05 and a log2-fold change ≥ 1. Duplicate samples were used for each condition.

### 2.16. Statistics

GraphPad prism V9 (GraphPad Software Inc., La Jolla, CA, USA) was used to analyze data. Data sets were compared for statistical significance using a two-tailed Student *t*-test whenever only two groups were being compared. For more than two groups, a parametric two-way analysis of variance (ANOVA) with Bonferroni’s correction was used if the data were found to fit a Gaussian distribution (tested using the method of Kolmogorov and Smirnov). When not normally distributed, the data were analyzed using a nonparametric two-way ANOVA with the Mann–Whitney U test. An α value of 0.05 was used to determine whether a significant difference existed between data from untreated control and experimental treatment groups.

## 3. Results

### 3.1. Generation of Lactococcus Lactis Expressing SlpA, a Novel Clinical Candidate for Treating IBD

**Figure 1:**
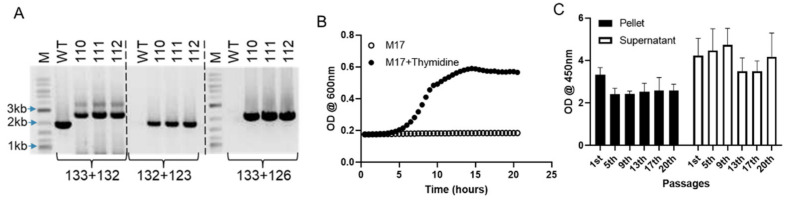
Generation of a SlpA-expression bacterial strain. (**A**) Primers 132 and 133 were used to screen for SlpA+ and Thy- colonies that amplify ~2 Kb amplicon in WT and ~2.6 Kb amplicon in SlpA+ integrated colonies; primers 132 and 123 are specific for integrated SlpA amplifies a gene specific amplicon. Primer sets 132 and 123, as well as 133 and 126 were used to further confirm the integrated *SlpA* in the colonies. Results represent PCR confirmation of three different clones. (**B**) Representative growth curve of clone R110 in M17 media with and without thymidine supplementation. Bacteria were incubated with the M17 media with and without thymidine supplementation in a 96 well plate, and growth was measured by OD660nm every 30 min for 20 h. (**C**) Stability of the SlpA cassette was demonstrated by culturing bacteria for 20 generations. Expression of SlpA was detected in the cell pellet and supernatants from passages 1st, 5th, 9th, 13th, 17th, and 20th by ELISA; experiment was run in triplicates; mean + SD.

To construct an acceptable SlpA delivery platform for human studies and improve the pharmacodynamic potential of SlpA, we developed an *L. lactis* probiotic expressing high levels of secreted SlpA from a genome integrated cassette. This R110 strain encodes a containment mechanism to control the genetically modified organism’s environmental growth after excretion, as the ThyA gene has been deleted and the strain requires supplementation with thymidine for growth. The R110 strain was generated by integrating the SlpA expression cassette into the *L. lactis* genome using double homologous recombination methods. We employed a proprietary plasmid (pRISE 1.2, Rise Therapeutics), which contains a temperature-sensitive origin of replication and the *SlpA* gene flanked by 500 bp sequences 5’ and 3’ of the thyA coding region [19,21]. When transformed into *L. lactis* and the growth temperature shifted to 37 °C, the SlpA expression cassette was inserted at the *thyA* locus via a double crossover event, replacing the *L. lactis thyA* from the chromosome. The purpose of targeting and deleting the *thyA* gene is to enable an already vetted containment strategy [20,22,23]; that is, when R110 is excreted, it will not propagate, due to the lack of thymidine. The *L. lactis* clones containing a stably integrated SlpA expression cassette were selected on minimal media supplemented with trimethoprim and thymidine, as previously described [24,25]. The stability of the integration event was confirmed by growing the clones under non-selective conditions for 100 generations. A PCR strategy was used to screen clones for proper *SlpA* gene integration. Three pairs of unique primers (Supplemental Table S1), representing sequences from either *slpA* or the flanking *thyA* untranslated regions, were used for the PCR screening (Figure 1A). 

To confirm that the SlpA cassette integration and protein expression did not alter the bacterium growth characteristics, the SlpA-expressing *L. lactis* clone R110 was grown in M17 medium with or without thymidine-supplementation and compared to the wild-type *L. lactis* (WT). Individual colonies were selected from freshly plated samples and the growth of each strain was followed in duplicate. The optical density of the culture was measured every 30 mins for 20 h. R110 demonstrated growth comparable to the WT *L. lactis* strain when the culture medium was supplemented with thymidine (Supplemental Figure S1). As expected, in the absence of thymidine, R110 did not grow (Figure 1B). The advantage of genome integration is to obtain a cell line that expresses the SlpA protein more stably and consistently from passage to passage. To confirm SlpA stable expression, R110 was subjected to continuous culture for 20 passages. Culture samples were taken at passages 1, 5, 9, 13, 17, and 20, cells were pelleted, and lysates were generated. SlpA protein expression levels were measured by a quantitative ELISA on both the cell lysate and supernatant (Figure 1C). R110 SlpA expression levels remained similar throughout the 20 passages tested, a feature of a stable clone. 

### 3.2. R110 Protects Mice from T Cell-Mediated Colitis

**Figure 2:**
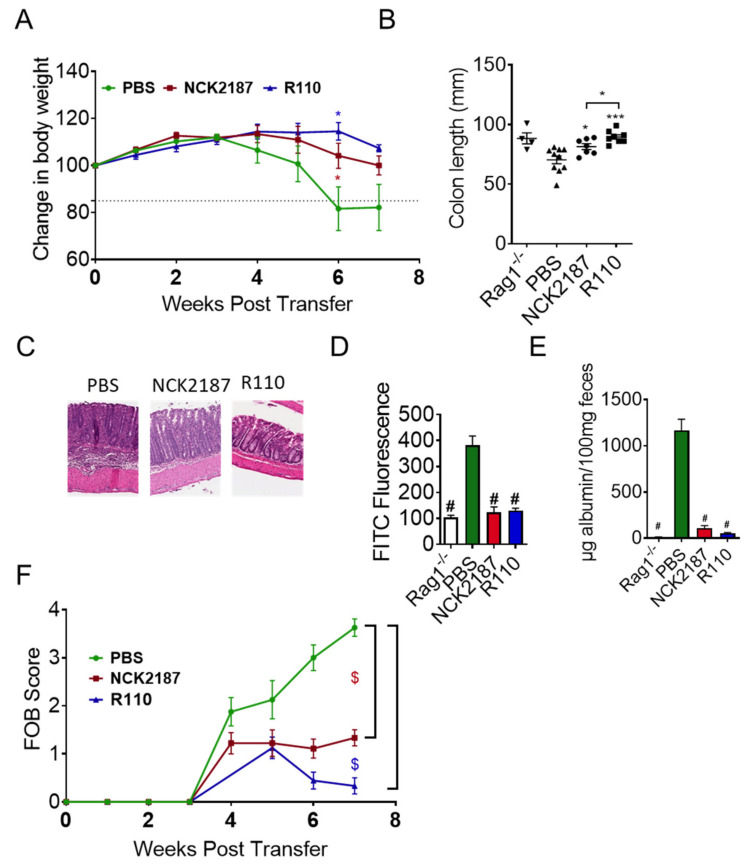
SlpA-expressing bacteria mitigate colitis. Rag1^−/−^ mice were injected with 5 × 10^5^ CD3^+^CD4^+^CD45RB^Hi^ T cells isolated from C57BL/6 mice and then orally gavaged with NCK2187 (red), R110 (blue), or PBS (green) three days before the transfer, and subsequently once a week for seven consecutive weeks. Colitis severity was determined, in part, by weight loss, gut permeability, FOB, colon length, and histopathology. (**A**) Each mouse was weighed every week, and the weight changes were depicted using the weight at the initiation of the experiment as 100 percent. (**B**) Colon lengths were measured at the end of the experiment. (**C**) The colon of mice was stained with hematoxylin and eosin to observe the structure and infiltrating cells. (**D**) Mice were fed with FITC-dextran, and four hours later serum from euthanized mice was tested for FITC fluorescence. (**E**) Serum albumin was measured in the fecal material at the end of the experiment. (**F**) Feces were collected every week to evaluate the fecal occult blood in the feces using a commercial kit. Data represent three individual experiments and are shown as mean ± SEM; *n* = 5–10 mice/group; * *p* < 0.05; # *p* < 0.001; *** *p* < 0.001; $ *p* < 0.0001.

We previously published that SlpA expressed on the surface of *Lactobacillus acidophilus* NCFM (NCK2187), protects mice from colitis [15]. We tested R110 in the T cell adoptive transfer model of colitis, which shares the pathophysiology of human Crohn’s disease. Rag1-deficient (^−/−^) mice were injected with 5 × 10^5^ naïve T cells (CD3^+^CD4^+^CD45RB^Hi^) to induce colitis. Mice were fed with 1 × 10^9^ CFU of freshly prepared R110 every week, starting three days before introducing naïve T cells. A group of mice were also provided with 1 × 10^9^ NCK2187 as a positive control and PBS as a negative control. Mice were weighed every week for eight weeks for the evaluation of the disease outcome, and feces were collected weekly to evaluate the fecal occult blood, as a measure of colitis severity (Supplemental Figure S2). Mice fed with R110 or NCK2187 resisted the weight loss (Figure 2A). In contrast, mice fed with PBS lost more than 15% of their initial weight (*p* < 0.05 compared to R110 and NCK2187) after six weeks, and some mice in this group succumbed (data not shown). Mitigation of colitis by SlpA was also seen at tissue level. Eight weeks after introducing naïve T cells, mice were sacrificed, and colon length was measured. During colitis, the colon shrinks and becomes fragile, due to infiltrating cells and ongoing inflammation. Upon measurement of the colon length, the treatment with R-110 maintained the colon length (Figure 2B). The histopathological evaluation of colonic tissues showed a sharp reduction in inflammatory cells in the colons of mice fed with R110 (Figure 2C), but not in PBS-treated mice. Overall, oral treatment with R110 significantly protected mice from colitis. Similar studies were carried out using the WT *L. lactis* line; however, the WT strain did not protect the mice from colitis-mediated weight loss (data not shown).

IBD patients display several defects in the many specialized components of the mucosal barrier, from the mucus layer composition to the adhesion molecules that regulate paracellular permeability [10]. At the end of eight weeks, four mice in each group were fed with FITC-dextran and euthanized four-hour post-gavage to collect serum for the detection of gut permeability by FITC dextran (Figure 2D). Feces were also tested for serum albumin levels to confirm gut permeability findings (Figure 2E). The results showed that oral the delivery of R110 significantly mitigated gut barrier leakage, as demonstrated by reduced FITC-dextran in the circulation and lower level of albumin in the feces. Similar results were seen with the positive control strain (NCK2187) but not in the PBS-treated mice. Inflammation and increased permeability also led to blood oozing in the feces, which was detected by measuring the hemoglobin in the feces using a fecal occult blood (FOB) detection kit (Figure 2F). The data showed that R110 significantly reduced the FOB, compared to the untreated mice (*p* < 0.05). 

### 3.3. R110 Induces Positive Changes in the Transcriptome of Colon Tissue

**Figure 3:**
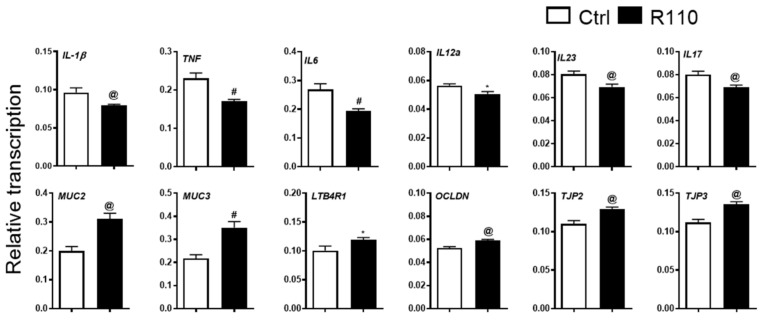
SlpA reduces local inflammation and leaky gut. Total RNA was isolated from the distal colon of mice from Rag1^−/−^ mice after eight weeks of T cell-mediated colitis initiation. Results represent means ± SEMs from three independent experiments; *n* = 7–12; * *p* < 0.05; @ *p* < 0.01; # *p* < 0.001.

We next assessed changes at the transcriptomic level. Two-millimeter-long distal colon pieces were excised, and total RNA was isolated and converted to cDNA for the detection of target genes (Figure 3). Transcripts from inflammatory cytokines, such as *IL-1β, TNF, IL-6, IL-12a, IL23*, and *IL-17* were significantly reduced in mice treated with R110 than in the control PBS-treated animals. The colon separates host tissue from the gut microbiota by the presence of mucins. The major mucins present in the colorectal area are mucin 1 (Muc1), mucin 2 (Muc2), mucin 3 (Muc3), and mucin 4 (Muc4). Out of these four mucins, Muc2 and Muc3 have been shown to play a significant role in a healthy colon [26,27,28]. In mice treated with SlpA-expressing bacteria R110, the expression of *Muc2* and *Muc3* where significantly increased, compared to the control. Occludin and tight junction proteins act as adhesives among the intestinal epithelial cells to aid in the barrier function. Additionally, in this case, *occludin* and *tight junction proteins 2* and *3* levels were significantly elevated by SlpA treatment. We also noted a similar increase in *LTB4R1* expression in mice treated with SlpA-expressing bacteria. LTB4R1 is essential for IgA production in the gut and acts as a protective means for gut microbial homeostasis. Overall, these data corroborate the protective role of SlpA in diminishing inflammation and restoring gut barrier integrity and extends our mechanistic understanding of the underlying molecular factors mediated by SlpA.

### 3.4. R110 Induces IL-10 and Reduces Rorγt and IL-17 Production

**Figure 4:**
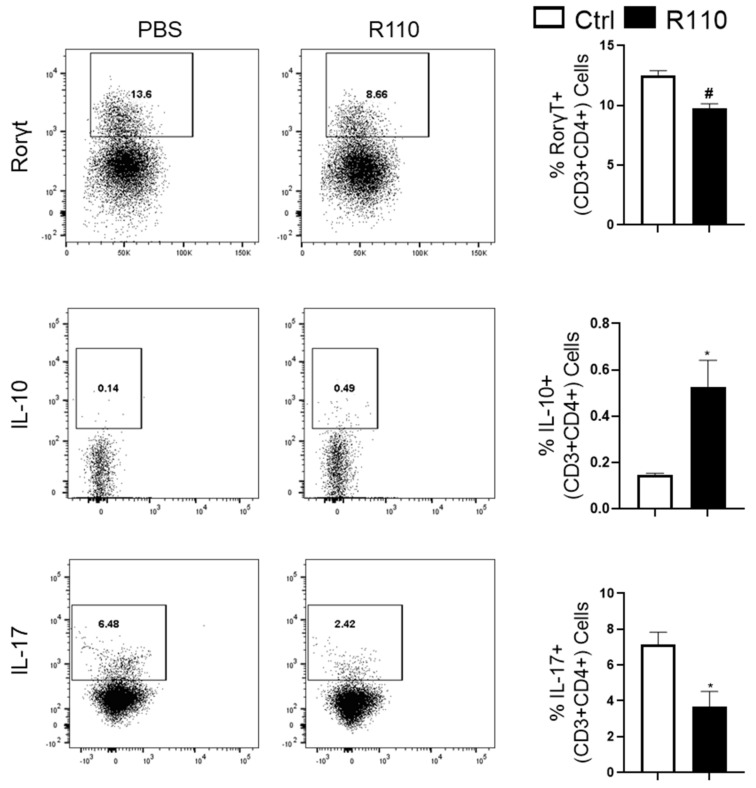
SlpA modulates the T cell response in colitis mice. Colonic cells isolated and stained for CD45, CD3, CD4, Rorγt, IL-17, and IL-10 from Rag1^−/−^ mice after 8 weeks post-initiation of colitis and the subsequent feeding of R110. Data are shown as mean ± SEM and are representative of three different experiments; *n* = 6 mice/group; * *p* < 0.05; # *p* < 0.001.

The observed transcriptomic changes suggest a role of SlpA in modulating the gut immune response. Therefore, we sought to understand the role of T cells in this model. Rag1-deficient mice were injected with one million CD45RB^Hi^ cells and then were treated with R110 or PBS once a week, as indicated before. Four weeks post-introduction of the T cells, mice were euthanized to collect the colonic lamina propria cells. The isolated cells were selected for CD4^+^ and then stained for Rorγt, IL-17, and IL-10 expression. CD4^+^ T cells isolated from mice treated R110 showed a significant decrease in inflammatory Rorγt and IL-17 producing T cells and an increase in anti-inflammatory IL-10 producing T cells, compared to control mice (Figure 4), further supporting the ability of SlpA to engage the immune system, leading to a diminution of inflammation.

### 3.5. R110 Reduces Critical Inflammatory Cytokines in the Sera of Diseased Mice

**Figure 5:**
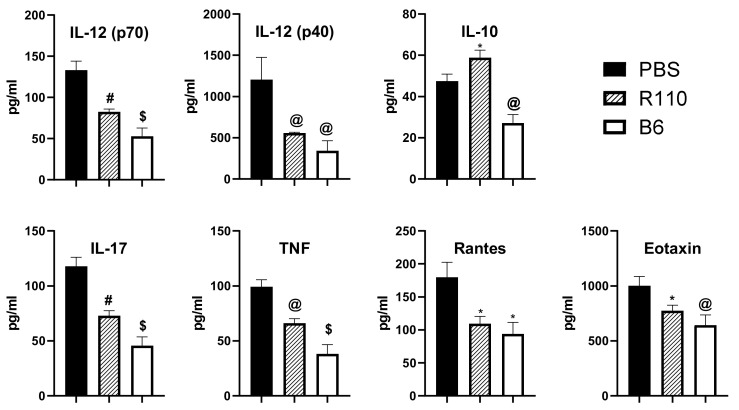
SlpA engages the gut immune cell to reduce systemic inflammation. Serum collected from mice from Rag1^−/−^ mice after 8 weeks of T cell-mediated colitis initiation. Serum from mice without colitis (B6) was used as reference. Results represent means ± SEMs from three independent experiments; *n* = 5–10 mice/group; * *p* < 0.05; @ *p* < 0.01; # *p* < 0.001; $ *p* < 0.0001.

We next assessed whether, in addition to local anti-inflammatory properties, R110 could also affect inflammation systemically. To test whether R110 reduces systemic proinflammatory cytokines, mice were euthanized after four weeks, post-introduction of naïve T cells. As additional control, the serum cytokines of Rag1^−/−^ treated mice were compared with serum cytokines of healthy WT C57Bl/6 (B6) mice. The sera were tested for 23 cytokines (Eotaxin, G-CSF, GM-CSF, IFN-γ, IL-1α, IL-1β, IL-2, IL-3, IL-4, IL-5, IL-6, IL-9, IL-10, IL-12 (p40), IL-12 (p70), IL-13, IL-17A, KC, MCP-1, MIP-1α, MIP-1β, RANTES, and TNF-α). The results showed that treatment with SlpA expressing bacteria significantly reduced the levels of proinflammatory cytokines (IL-12 (p70), IL-12 (p40), IL-17, TNF, Eotaxin, and RANTES) and increased levels of IL-10 (Figure 5). 

### 3.6. R110 Protects the Gut Microbiota

**Figure 6:**
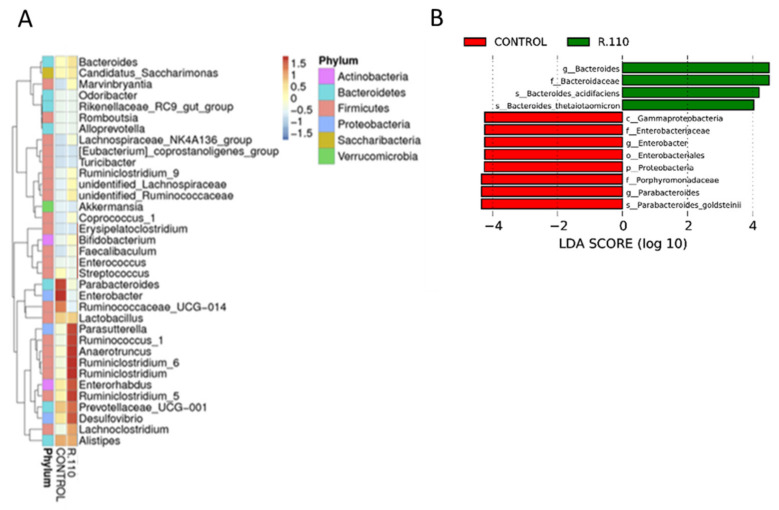
Protection of the gut microbiome by SlpA. Fecal DNA was isolated from Rag1^−/−^ mice 8- weeks after T cell-mediated colitis initiation. Amplicons were generated using specific primers for V3 and V4 regions of the 16sRNA gene. The amplicon was sequenced using an Illumina high-throughput sequencer with a paired-end sequencing strategy. (**A**) Phylum-wise genera were clustered to show the differential accumulation of bacterial genera in the different treatment groups. (**B**) Linear discriminant analysis (LDA) shows the genus and species of the bacteria that are differentially present between the mice fed with R110 or PBS; *n* = 5–7 mice/group.

Microbiota harboring the gut plays a critical role in health and disease. We collected feces from colitis mice that were treated with R110 or PBS, with daily oral administration, for a month. Total DNA was isolated and the V3–V4 region of the bacterial DNA was sequenced using amplicon sequencing. As expected, the results showed a decrease in bacteria relative abundance in control mice, which was mitigated by R110 administration, suggesting a protection of the gut microbiome richness (Figure 6). In addition, the administration of R110 favored taxa that are associated with gut health. The most significant changes were found in six major phyla: Actinobacteria, Bacteroidetes, Firmicutes, Proteobacteria, Saccharibacteria, and Verrucomicrobia. Mice were treated with R110 enriched bacteria belonging to families Parasutterella, Ruminicoccus, Anaerotruncus, Ruminiclostridium, Enterorhabdus, Prevotellaceae, Desulfovibrio, and Lachnoclostridium (Figure 6A). At a species level, a linear dimensional analysis (LDA) revealed that R110-fed mice were enriched in two bacterial species, *Bacteroides acidifaciens* and *Bacteroides thetaiotamicron* (Figure 6B).

### 3.7. R110 Induces Regulatory Cytokines in Human Dendritic Cells

**Figure 7:**
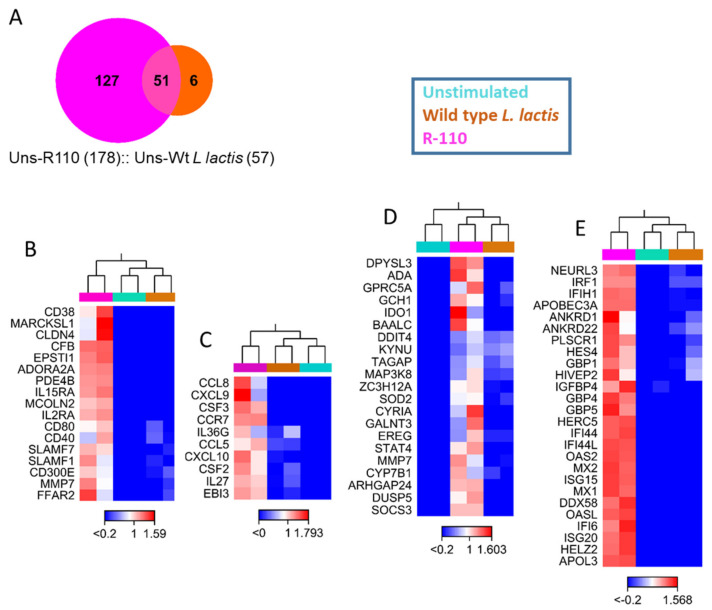
SlpA engages human dendritic cells to trigger differential gene expression. Human dendritic cells were stimulated with R110, WT *Lactococcus lactis* strain, or left unstimulated. Total isolated RNA were sequenced using Novaseq at a commercial site. The transcriptomic data were analyzed using CLC Genomic Workbench v. 20. Differential expression genes were determined with a selection threshold of *p*-value ≤ 0.05 and log2-fold change ≥ 1. Duplicate samples were used for each condition. (**A**) Ven diagram showing the numbers of differentially expressed genes at different stimulation. (**B**–**E**) Heat maps showing the differential gene expression of cell surface receptors, (**B**) cytokines and chemokines, (**C**) immunomodulating enzymes, (**D**) and interferon-related genes (**E**) in response to R110.

R110 has shown striking efficacy to mitigate colitis in mice. To investigate the translational potential of R110, we cocultured human monocyte-derived dendritic cells with the WT *L. lactis* strain or R110 at the ratio of 1 for 12 h. Total RNA was isolated from these samples and sent for the entire exome RNA sequencing. The three groups (unstimulated, stimulated with WT, or R110) were compared against each other and among themselves. WT *L. lactis* stimulated a total of 57 genes in dendritic cells, whereas R110 stimulated 178 genes; 51 genes were common, which were the genes stimulated by *Lactococcus* (Figure 7A and Supplemental Figure S3A). Six genes were only stimulated by the WT strain and not by R110 (Supplemental Figure S3B). The 127 genes that were specifically stimulated by the R110 included 74 coding genes and 53 noncoding genes. The 74 genes were grouped in four primary categories: cell surface receptors (*CD38, CD40, CD80, IL-15RA, IL-2RA, CD300e, CCR7,* and *FFAR2*), cytokine (*IL-27, IL36g, EBi3, CSF, GM-CSF*, and *LTA*), chemokines (*CCL8, CXCL9, CCL5,* and *CXCL10*), enzymes (*ADA, SOCS3, SOD2, KYNU, IDO1,* etc.), and antiviral genes (*IRF1, IFI44, IFI44L, OSA2, OSAL, ISG15, MX1*, *APOBEC3A, GBP4, GBP5*, etc.). We also observed an increase in 18 noncoding RNAs, which may have a broader impact on dendritic cell behavior. The WT *L. lactis* induced 6 genes, *AC025034.1, DUSP1, HCAR2, HCAR3, RGS1*, and *GPR183*. The expression of some of these genes was confirmed by real time PCR (data not shown). A cytokine of interest, unveiled by this experiment, was IL-27, which was then investigated further.

### 3.8. R110 Induces IL-27 in Murine Dendritic Cells

**Figure 8:**
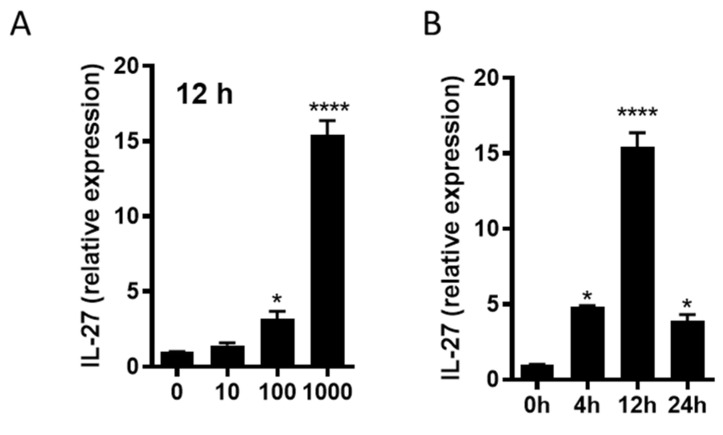
SlpA leads to increased expression of IL-27 in murine dendritic cells. Murine bone marrow cells were differentiated into dendritic cells using GM-CSF. (**A**) Murine dendritic cells were incubated with increasing concentration of purified SlpA (0, 10, 100, and 1000 ng/ml) for 12 h. (**B**) Murine dendritic cells were exposed to 1000 ng/ml of purified SlpA for 4, 12, and 24h. Total RNA was isolated and converted to cDNA and used as template for the evaluation of the IL-27 transcript. GAPDH was used as endogenous control; *n* = 3; * *p* < 0.05; **** *p* < 0.0001.

**Figure 9:**
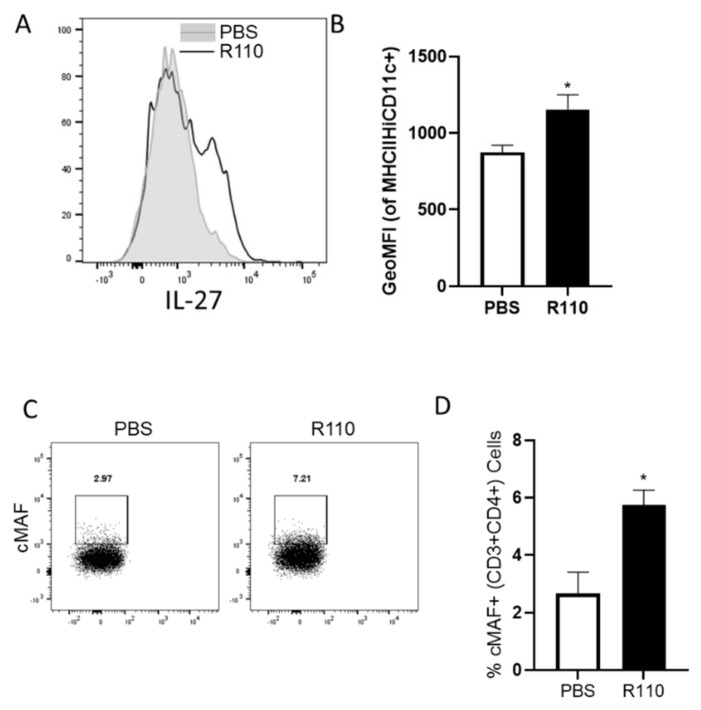
Oral administration of R110 increased IL-27 in colonic dendritic cells and c-MAF in T cells. Rag1^−/−^ mice were introduced with the 5 × 10^5^ naive CD4 T cells to induce colitis. Mice were gavaged with R110 every week for a month. Colonic dendritic cells (CD45^+^CD11c^+^MHC^Hi^) were analyzed for IL-27 production. (**A**) Histogram showing the expression of IL-27 in control-derived dendritic cells (filled graph) or R110-derived dendritic cells (open graph). (**B**) Geometric mean of the fluorescence for PE channel representing IL-27. (**C**) Colonic CD4 T cells stained for cMAF dot plots. (**D**) bar diagram showing the percentage of CD4 T cells expressing cMAF; *n* = 5 mice/group; * *p* < 0.05.

We further evaluated the transcription of IL-27 in bone marrow derived dendritic cells in response to the SlpA. Murine dendritic cells were generated from the isolated bone marrow cells and incubated with purified SlpA protein at increasing concentrations. Total RNA was isolated from the dendritic cells after 12 h post-incubation and subjected to quantitative real time PCR using 18sRNA as an internal control. We observed a dose-dependent increase in IL-27 transcript in the cells exposed to SlpA (Figure 8A). To understand the kinetics of IL-27 transcription, we repeated the experiment using the highest concentration of purified SlpA protein (1000 ng/ml). IL-27 transcription levels accumulated in a time-dependent fashion for up to 12 hours (Figure 8B). To assess whether these results also held true in vivo, we gavaged colitis-induced mice with R110, as earlier, for four weeks, and then IL-27expressing dendritic cells (CD45+CD11c+MHCIIHi) were measured in colonic cells. We detected significantly elevated IL-27 expression in the colonic dendritic cells in mice fed with R110, compared to untreated mice (Figure 9A,B). The heterodimeric receptor for IL-27 (IL-27R) is expressed on a variety of cells, including T cells. In the same experiment we also evaluated cMAF, a transcription factor that is expressed upon the IL-27 signaling and controls the behavior of T cells by suppressing Th17 and augmenting Tregs and IL-10 production. In mice fed with R110, the expression of cMAF on the T cells was increased (Figure 9C,D), suggesting IL-27 signaling on these cells.

## 4. Discussion

IBD is a chronic inflammation of the GI tract and a global health concern. The disease is more prevalent in Western countries, but it is rapidly increasing in the newly industrialized world. The etiology of IBD is complex and may vary significantly in the population. Despite the wide range of causative agents, the disease is characterized by inflamed tissue and dysbiotic microbiota. Dampening the inflammation and re-establishing a healthier gut microbiome have been shown to influence each other, leading to improvement in the disease severity. For example, anti-TNF therapy reduced ongoing inflammation, promoting a healthier gut microbiota [29]. Anti-cytokine treatment is often used in IBD patients; however, their rate of failure is gradually increasing, due to the development of anti-drug antibodies, and possible opportunistic infections put the patient at risk [30]. A new and alternative approach to treating IBD is to modulate the gut microbiota. A meta-analysis suggested antibiotics as an effective therapy against IBD, mainly by the elimination of dysbiotic microbiota. However, antibiotic treatment also eliminates the chances of reinstating a healthy microbiota and provides opportunity for the outgrowth of harmful bacteria, such as *Clostridium difficile*. An alternative to antibiotic therapy is FMT, where few clinical trials have shown FMT benefits. However, FMT is fraught with safety issues that hinder its use, such as the potential risk of transferring antibiotic-resistant bacteria [11,12]. Probiotics have also been used with mixed results. Previously, we and others identified the role of surface layer protein A (SlpA), expressed in *L. acidophilus*, in mitigating gastrointestinal inflammation [15,16,17]. Given the potential therapeutic role of SlpA, we engineered a food-grade probiotic, *Lactococcus* (*L.) lactis*, to express SlpA. This strain, R110, is optimized for the pharmacodynamic delivery of SlpA via a secreted mechanism. In addition, the SlpA-expressing cassette was inserted by replacing the *thyA* gene, hence providing a containment strategy, such that the organism will not grow after being excreted from the body. The disruption of *thyA* has been a standard for biological containment strategy to prevent the environmental dissemination of an engineered live vaccine or drug delivery vehicles in the past [20,31,32]. Such modified *Lactococcus* strains have already been used in human trials [31,32]. In this paper, we confirmed the pivotal role of SlpA in mitigating colitis and unveiled a new understanding of the mechanism by which SlpA mediates its anti-inflammatory effects.

An important SlpA mechanism of action is its role in protecting the gut epithelium. The mucus layer plays a critical role in maintaining the microbiota in the gut lumen by preventing immune stimulation, due to their recognition by immune cells. The three primary mechanisms used by the gut epithelial layer are: (i) release of antimicrobial peptides, (ii) release of mucus, and (iii) paracellular permeability. The mucus layer is composed of different mucin protein types; some are transmembrane, and others are secreted by the specialized epithelial cells, the goblet cells. MUC2 and MUC3 are the two important mucins that are present in the intestines, and their expression is reduced during colitis [26,33,34]; mice deficient in *Muc2* develop spontaneous colitis [28]. An increase in the transcription levels of *Muc2* and *Muc3* represents a mechanism of action by which SlpA provides protection against inflammation and protects the gut epithelium. To prevent any serum protein leakage, the gut epithelial layer maintains extremely low paracellular permeability, which is determined by the complex structures of tight junctions located between the epithelial cells. These tight junctions are made up of transmembrane proteins, such as occludins, junctional adhesion molecules (JAM), and claudins, with an intra-cellular connection to the zonulins, which are members of the zonula occludens (ZO) family [35]. As predicted from the gut barrier results, we found an increase in tight junction transcripts in the mice gavaged with R110. Moreover, the sharp reduction in the detection of fecal albumin and FITC dextran confirmed the physiological importance of these SlpA-mediated changes in membrane barrier activity. The mechanism by which SlpA can upregulated tight junction expression and mucin production is currently under investigation. Collectively, SlpA can exquisitely lead to the upregulation of gut barrier integrity, which represents one mechanism of action of this protein. A second novel mechanism of action is the upregulation of IL-27 by SlpA. IL-27 has been demonstrated to play an important role in inflammation-related disease. We demonstrated in both mouse in vitro stimulation assay, as well as in vivo study, an increased expression of IL-27, making IL-27 a signature cytokine for SlpA response. IL-27 has variety of effects on immune responses, and it is now well-recognized as a potent antagonist of different classes of inflammation, through its ability to modify T cell effector functions directly, induce IL-10, and promote specialized T regulatory cell responses [36]. IL-27 induces the transcription factor cMAF, which stabilizes the FoxP3 in Tregs [37,38], induces IL-10 in a variety of T cells [39,40,41,42], and promotes IgA secretion from B cells [38]. Mice fed with R110 showed an increase in cMAF positive T cells, suggesting that active IL-27 signaling in these mice may be responsible for the SlpA beneficial effect. The upregulation of IL-27 was also confirmed in human dendritic cells, suggesting a shared mechanism among species, and therefore, the high likelihood that R110 would be beneficial in humans with IBD.

The therapeutic effects of SlpA were exerted by diminishing inflammatory mediators and pro-inflammatory CD4^+^ T cells. Interestingly, the local effects seen in the GI tract were also observed systemically. One cytokine that was downregulated was IL-12(p70),a classical cytokine determining the activation of Th1 cells, and IL-12(p40),which could contribute to IL-23 and IL-12 (p80) generation. IL-23 plays a key role in Th17 cell development, whereas IL-12(p80) helps in the migration of myeloid cells and potentiates IFNγ release by Th1 cells [43]. TNF and IL-17 expression were also reduced by SlpA. TNF is a potent inflammatory cytokine contributing to the pathology of IBD and inhibition of TNF signaling and has been a vital alternative to treat patients; IL-17 secreting T cells have been shown to contribute to the immunopathology in IBD [44]. A downregulated chemokine was RANTES, which is involved in the granulomatous structure in IBD-affected intestines and whose levels are elevated in IBD patients [45]. 

Interestingly, the effects of SlpA on dendritic cells were not solely anti-inflammatory. Instead, it was a mixed response, where some adhesion molecules and chemokine receptors were also elevated. Some inflammatory gene signatures were also noticed, and a big group of interferon-related genes were transcribed. This represents a new mechanism of action unveiled in this study. Dendritic cells exposed to SlpA enhanced the adenosine deaminase expression, which is a cell surface receptor that maintains the activation of dendritic cells. CCR7 transcripts were also elevated and are required for dendritic cells to respond to CCL19 and CCL21. Two costimulatory molecules that were found to be more prevalent were CD40 and CD80 [46]. A higher expression of CD40 leads to the differentiation of Th1 cells [47]; however, certain levels of CD40 are still required on dendritic cells for the generation of Treg, as CD40-deficient mice carry fewer numbers of Treg in the periphery [48]. CD80 interacts with CD28 and CTLA4 on T cells, and the absence of CD80 interactions lessens the suppressive effects of Tregs, as well [49]; however, CD80 also contributes to controlling Th1 cell proliferation [50]. Along with CD40 and CD80, two more CD molecules elevated by SlpA were CD300e and CD38. CD300e belongs to a more prominent family of molecules, CD300, with six known members, namely CD300a-f. Members of CD300 interact with different endogenous and exogenous lipids for their activation, such as cardiolipin A, lipid A, phosphatidic acid, and phosphatidylcholine. CD300e engagement enhances activation markers’ expression, including CD40, CD80, and CD25, with a concomitant reduction in proinflammatory cytokines [51]. CD38 is a surface-bound enzyme with nicotinamide adenine dinucleotide (NAD) as a substrate [52]; CD38 deficiency increases susceptibility to various bacterial pathogens [53,54,55]. Additionally, it is considered a robust M1 macrophage marker [56]. Two short-chain fatty acid receptors were also elevated, FFAR2 (or GPR43) and GPR132. GPR132 is a receptor expressed in tumor-associated macrophages [57,58,59], and the engagement of GPR132 attenuates inflammatory cytokines [60]. In addition, GPR132 senses lactate in the environment, which may favor the presence of beneficial lactate-producing bacteria. FFAR2 is well-studied in gastrointestinal diseases. It is a receptor for acetic, propanoic, butyric, and pentanoic acids. Several papers have been published describing the protective role of FFAR2 on various cells, including the upregulation of Amphiregulin [61] and secretion of IgA [62]; a recent paper was published by Garrett’s laboratory elucidating the role of this receptor on dendritic cells. FFAR2 deficient dendritic cells lead to exacerbated colitis by controlling excessive IL-27 [63]. Since SlpA-induced IL-27 expression, FFAR2 may counteract this signaling, preventing excessive high levels of IL-27, which could be detrimental. Similarly, we noticed the upregulation of ADORA2A and SLAMF7, which can reduce excessive T cell activation, suggesting a direct regulatory control exerted by SlpA. Overall, SlpA appears to favor a balanced immune response. 

Another important novel mechanism of action was on the gut microbiome. R110 administration protected the gut microbiome by favoring a higher relative abundance of species, compared to control mice. In addition, R110 favored the growth of beneficial bacteria. The microbiome of R110-fed mice was enriched with Proteobacteria (*Parasutterrella* and *Desulfovibrio*), Actinobacteria (*Enterorhabdus*), and Firmicutes (*Ruminococcus*, *Anaerotruncus*, *Ruminiclostridium*, and *Lachnoclostridium*). *Parasutterella* and *Anaerotruncus* are common gut bacteria that produce succinate [64,65], which is an intermediate for the production of propionate and butyrate by other microbial organisms, such as *Bifidobacteria* and *Bacteroides thetaioatamicron* [66,67]. Succinate acts as an anti-inflammatory molecule in the gut [68]. *Enterorhabdus* has been showed to protect mice from colitis [69]. *Desulfvibrio* belongs to *Proteobacteria*, and it is enriched in mice that resisted the colitis [69]. Bacteria from family *Lachnospiraceae* and order *Clostridiales* were also increased in R110 fed mice. Both of these bacterial groups are butyrate-producing and prevent inflammation and colitis [70,71,72,73,74,75]. Moreover, two bacterial species were specifically enriched in R110-fed mice, *Bacteroides* *thetaiotamicron* and *Bacteroides acidfaciens*. *B. thetaiotamicron* promotes intestinal villus vascularization [76], restores fucosylation [77], stimulates the production of antimicrobial peptides [78], and competes with pathogenic bacteria for food sources [79]. *B. acidfaciens* is overrepresented in mice resistant to colitis [69,80], prevents IgE receptor expression on mast cells [81], enhances IgA production [82], assists in tumor killing [83], produces vitamin B6, and prevents the colonization of pathogenic bacteria, such as *Salmonella* [84]. Additionally, it enriched lean mice with healthy lipid profiles and improved insulin sensitivity [85,86,87]. Overall, SlpA favors the growth of bacteria in the gut that support a healthy microbiome with potential reduction of inflammation. Overall, these data confirm the gut microbiome as a central hub for human health, and the unique mechanism of the action of SlpA in dampening host inflammation. 

The mammalian intestine harbors trillions of bacterial species with a mutualistic relationship with the host. These bacteria help in the digestion and absorption of food; additionally, they synthesize various bacterial products, including essential vitamins. Inheritably, bacteria possess microbial-associated molecular patterns (MMPs), which would elicit a robust immune response upon recognition. Commensal bacteria either secrete or contain molecules on their surface that could modulate the immune function of host cells for a tolerogenic environment. Bacterial metabolites, such as short-chain fatty acids [67], indole [88], polysaccharides [69,89], and proteins [14] are the significant effectors that modulate host responses. Among proteins, several pathogenic and commensal bacteria products have shown immunomodulatory properties [14]. These proteinous bacterial products either (i) directly inhibit cellular signaling, such as a protein from *Yersinia pestis* (YoPM) [90], (ii) engage immune receptors, such as SlpA from *Lactobacillus acidophilus* [15] and colonization factor antigen 1 (CFA1) from *Escherichia coli* [91], or (iii) exhibit enzyme activity to modulate peptidoglycan, such as the SagA protein of *Enterococcus faecium* [14]. The effect of SlpA is closer to CFA1, where both are engaged in the generation of Tregs and IL-10 producing T cells to counterbalance the impact of IL-17, producing inflammatory T cells. However, CFA1 is known to mount its activity by the central cytokine IL-35 [91], whereas we found IL-27 is the strong signature for SlpA activity. Both of these cytokines are similar but different in their effects. IL-35 is completely anti-inflammatory, whereas IL-27 possesses both anti- and pro-inflammatory activity [92]. Future studies will help determine when SlpA-induced IL-27 aids existing inflammation or if it always tames it. Additionally, studies are in the process of evaluating the systemic effects of SlpA to prevent inflammation at extra-intestinal locations and pinpoint the mechanism(s) of action of this protein by using different murine models. 

In this study, we discovered a unique strategy utilized by SlpA, which targets the host immune cells to re-set the inflammatory repertoire. This could represent a survival strategy for bacteria carrying SlpA; that is, by making the host ‘healthier’, the engrafted bacteria have a higher chance of survival in the host gut microbiome. We created an environmentally restricted *L. lactis* line that expresses SlpA, which protects mice from T cell-induced colitis, by the induction of IL-10 and IL-27, restoration of gut epithelial barrier, reduction of local and systemic inflammation, protection of the gut microbiome, and the overall resetting of the host immune response. The new SlpA-expressing bacterial strain, R110, represents an important new clinical candidate that merits patient testing for the treatment of IBD. 

## Data Availability

Raw RNAseq data files used to generate Figure 7, submitted to NCBI as Bioproject ID # PRJNA758593 and Submission ID # SUB10279795.

## Supplementary Materials

The following are available online at https://www.mdpi.com/article/10.3390/biomedicines9091098/s1, Table S1: list of primers used in this study; Figure S1: representative growth curve of WT *L.latis* clone in M17 media with and without thymidine supplementation; Figure S2: a graphical representation of the mice experiment; Figure S3: human dendritic cells were stimulated with R110, *Lactococcus lactis* strain at ratio of 1, or left unstimulated.
