# Supplementary material for: Lactococcus lactis Delivery of Surface Layer Protein A Protects Mice from Colitis by Re-Setting Host Immune Repertoire
Source: Biomedicines. 2021 Aug 29;9(9):1098. doi: 10.3390/biomedicines9091098 (PMC8470720; doi:10.3390/biomedicines9091098)
Supplement: Supplementary file 1 [file biomedicines-09-01098-s001.zip › biomedicines-1333463-supplementary.pdf]

Supplementary Materials:

Gene	Sequence	Application	Reference
IL-1b	Forward: AAGGAGAACCAAGCAACGAC	qPCR	Lightfoot *et al.*, 2015
	Reverse: GAGATTGAGCTGTCTGCTCA	qPCR	
IL-17	Forward: ACTACCTCAACCGTTCCACG	qPCR	This study
	Reverse: TTCCCTCCGCATTGACACAG	qPCR	
PTGS2	Forward: CATCCCCTTCCTGCGAAGTT	qPCR	This study
	Reverse: CATGGGAGTTGGGCAGTCAT	qPCR	
Muc2	Forward: GTGTGGGACCTGACAATGTG	qPCR	Lightfoot *et al.*, 2015
	Reverse: ACAACGAGGTAGGTGCCATC	qPCR	
Muc3	Forward: GCCGTGAATTGTATGAACGGA	qPCR	Lightfoot *et al.*, 2015
	Reverse: CGCAGTTGACCACGTTGACTA	qPCR	
LTB4R1	Forward: ATGGCTGCAAACACTACATCTCCT	qPCR	Lightfoot *et al.*, 2015
	Reverse: CACTGGCATACATGCTTATTCCAC	qPCR	
OCLDN	Forward: GCTGTGATGTGTGTGAGCTG	qPCR	Lightfoot *et al.*, 2015
	Reverse: GACGGTCTACCTGGAGGAAC	qPCR	
TJP2	Forward: ATGGGAGCAGTACACCGTGA	qPCR	Lightfoot *et al.*, 2015
	Reverse: TGACCACCCTGTCATTTTCTTG	qPCR	
TJP3	Forward: TCGGCATAGCTGTCTCTGGA	qPCR	Lightfoot *et al.*, 2015
	Reverse: GTTGGCTGTTTTGGTGCAGG	qPCR	
18s RNA	Forward: ATAGCTGTATATTAAAGTTG	qPCR	Sahay *et al.*, 2009
	Reverse: GTCCTATTCCATTATTCC	qPCR	
R110	Forward: AATGCAACCATAACTGCCGC	qPCR	This study
	Reverse: TGTTGCGCCTGAGGTAACAT	qPCR	
NCK2187	Forward: CCAGCTATTGCCGGTAACCTT	qPCR	This study
	Reverse: AACGGCAGTAGTGCTACCAG	qPCR	
123	GCATGCTCGAGTCTAGAAGTCTTATAACTATAC	PCR	This study
126	GTCGACCGGTTTATCGAAAATTAGCTACTTTTAC	PCR	This study
132	GACCGCCTTTCCGTTATTATCC	PCR	This study
133	TGGGGCGGAAGTGGAAATACTTATGG	PCR	This study

Supplemental Table S1: List of primers used in this study

Supplementary Materials:

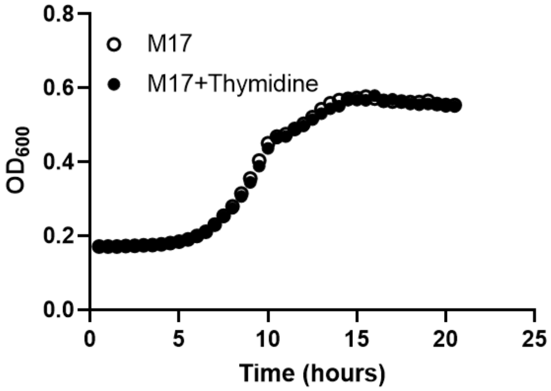

Supplemental Figure S1: Representative growth curve of wild type *L. lactis* clone in M17 media with and without thymidine supplementation. Bacteria were incubated with the M17 media with and without thymidine supplementation in a 96 well plate, and growth was measured by OD600nm in every 30 minutes for 20 hours.

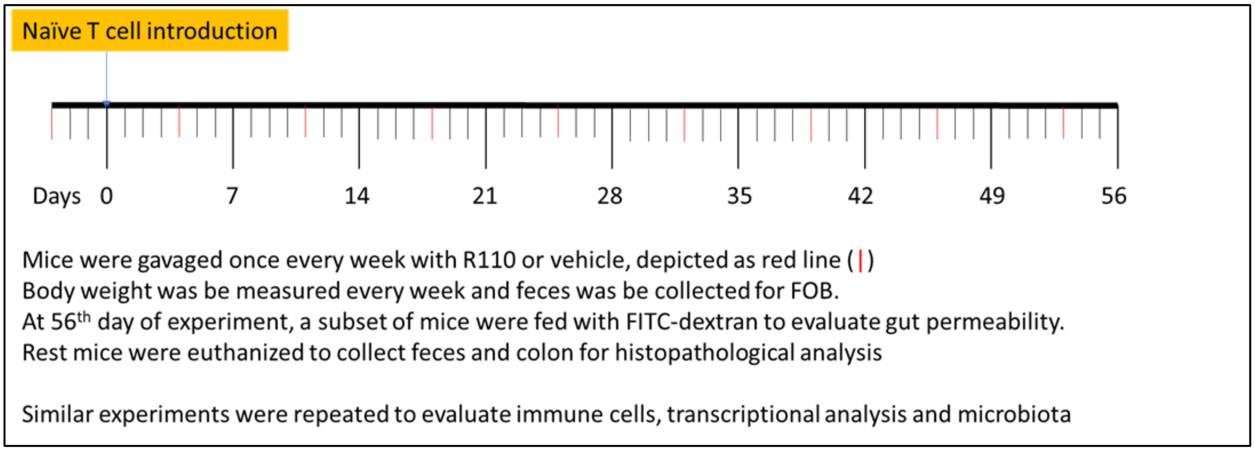

Supplemental Figure S2. A graphical representation of the mice experiment. Rag1$^{-/-}$ mice have injected with 5×10^5 naïve CD4 T cells (CD3$^+$CD4$^+$CD45RB^{Hi}) three days post-initiation of R110 or vehicle. Later, mice were fed with 1 billion CFU of R110 every week, as depicted in the red line. Mice were weighed every week, and feces were collected for FOB scoring. At the end of the experiment, a subset of mice was fed with FITC-dextran to observe its presence in feces as a measure of gut barrier integrity. The rest of the mice were euthanized to collect tissues and serum for analysis. Similar experiments were repeated to measure immune cells in lamina propria and microbiota analysis.

Supplementary Materials:

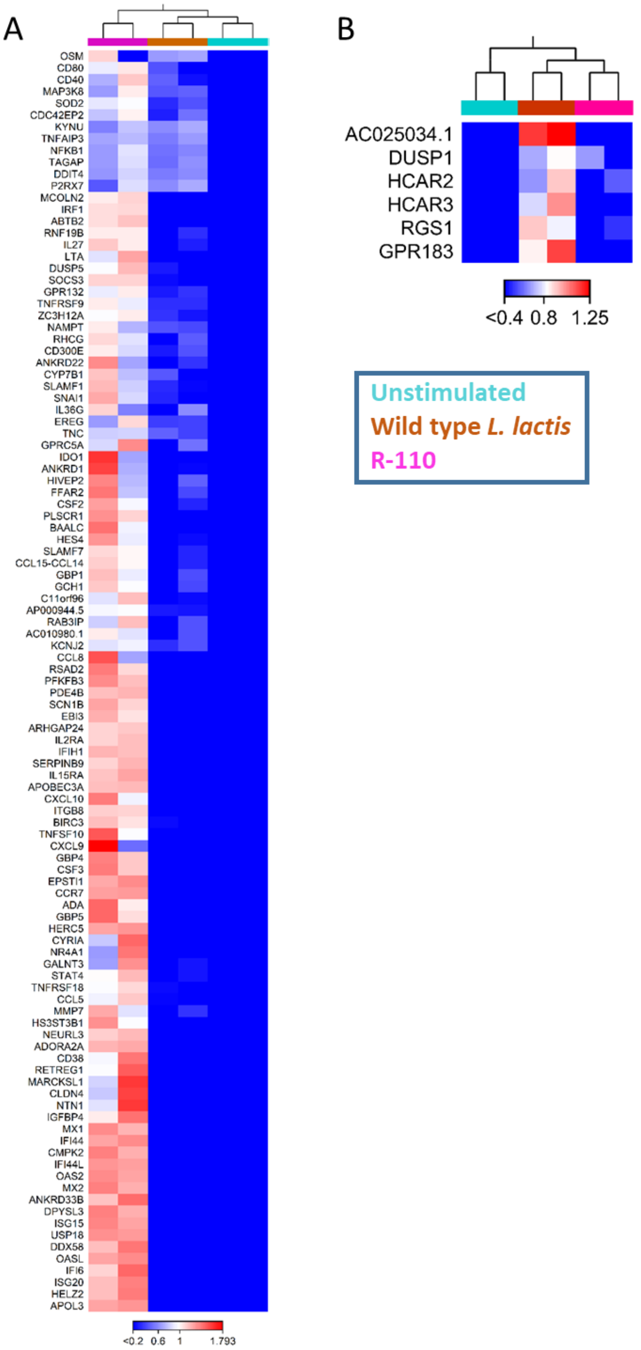

Supplemental Figure S3: Human dendritic cells were stimulated with R110, *Lactococcus lactis* strain at ratio of 1, or left unstimulated. Total isolated RNA was sequenced using Novaseq at a commercial site. The transcriptomic data were analyzed using CLC genomic workbench V 20. The data showed all the unique genes differentially expressed in DC exposed to R110 (A) and six genes that only expressed in DC exposed to wild-type *Lactococcus lactis*. Duplicate samples were used for each condition.
